# Silodosin improves functional consequences of lower urinary tract obstruction secondary to benign prostate hypertrophy, a proof of concept study in the spontaneously hypertensive rat supplemented with testosterone

**DOI:** 10.1186/s12894-020-00699-y

**Published:** 2020-08-27

**Authors:** Rana Assaly, Julie Faugeroux, Miguel Laurin, Sandrine Compagnie, Laurent Alexandre, François Giuliano, Delphine Behr-Roussel

**Affiliations:** 1 Pelvipharm, Montigny-le-Bretonneux, France; 2 Université Paris-Saclay, UVSQ, Inserm, END-ICAP, Versailles, France; 3 AP-HP, Neuro-Uro-Andrology, Department of Physical Medicine and Rehabilitation, Raymond Poincaré Hospital, Garches, France

**Keywords:** Benign prostatic hyperplasia, Lower urinary tract obstruction, Bladder function, Silodosin, Experimental cystometry, Spontaneously hypertensive rats

## Abstract

**Background:**

The main purpose of this study is to investigate the effect of silodosin on the urodynamic consequences in a previously established model of lower urinary tract symptoms associated with benign prostate hyperplasia, the spontaneously hypertensive rats (SHR) supplemented with testosterone.

**Methods:**

Three groups of animals (8-week-old; *n* = 10/group) were considered: Wistar Kyoto (control) rats (WKY), SHR supplemented with testosterone at 3 mg/kg/day and treated with either vehicle (SHR-T, *n* = 10) or silodosin at 0.1 mg/kg/day (SHR-T + silodosin, n = 10) by oral gavage for 6 weeks. Cystometry experiments were performed. The bladder was harvested, weighed and paraffin-embedded for morphometric analysis. The prostate was also harvested and weighed.

**Results:**

The number of animals included in the analysis were *n* = 10/10 for WKY and *n* = 7–8/10 for each SHR rats supplemented with testosterone group. SHR-T displayed a significant decrease in the intercontraction interval, infused volume and mean flow rate whereas the frequency of non-voiding contractions was increased. Silodosin improved the voiding behavior of SHR-T by significantly increasing the intercontraction interval, the infused volume and the mean flow rate and decreasing the number of non-voiding contractions. SHR-T displayed a significant increase in prostate and bladder weights and a 15% increase in the detrusor wall area compared to WKY.

**Conclusions:**

Chronic silodosin treatment relieved storage symptoms in SHR supplemented with testosterone and decreased the frequency of non-voiding detrusor contractions during the filling phase.

## Background

Lower urinary tract symptoms (LUTS) resulting from benign prostatic hyperplasia (BPH) are common problems in the aging male population [1]. These symptoms comprise two main components, the storage urinary symptoms also defined by the term overactive bladder and the voiding symptoms i.e. impairment of outflow.

According to guidelines and recommendations α1 receptor antagonists are first line drug treatment for patients with BPH/LUTS [2]. Among the subtypes of α1 receptors, it has been assumed that the 1A subtype is responsible for the dynamic component of BPH/LUTS and related voiding symptoms since it predominates in smooth muscle of prostate and proximal urethra [3]. Thus, the conventional mechanism of α1 receptor antagonists to relieve the symptoms is via relaxation of prostatic and urethral smooth muscle. In recent years, α1A receptor antagonists were reported to improve not only voiding but also storage symptoms. Indeed, several authors described changes in urodynamic parameters in BPH/LUTS patients suggesting a role for α1 receptor antagonists to decrease detrusor pressure at maximum urinary flow and to improve the degree of bladder outlet obstruction [4, 5]. Chapple et al [6] reported that silodosin, a uroselective α1A receptor antagonist, improved the International Prostate Symptom Subscores for both voiding and storage symptoms in LUTS/BPH patients. However, the mechanisms of action by which α1A receptor antagonists improve storage symptoms remain unclear. It has also been hypothesized the beneficial effects of α1A receptor antagonists may involve sites of action in addition to prostatic and urethral smooth muscle, such as the bladder and/or the spinal cord [7]. However bladder outlet obstruction remains a key pathophysiological feature in BPH/LUTS patients. In order to evaluate the effect of α1A receptor antagonists on voiding and storage symptoms associated with bladder outflow obstruction and to elucidate the corresponding mechanisms of action, it is important to use an animal model presenting urodynamic changes probably associated not only with mechanical obstruction caused by prostate enlargement but also with increase in urethral and prostate smooth muscle tone.

The present study was designed to investigate the effect of silodosin on the urodynamic consequences in a previously established model of LUTS related BPH [8], the spontaneously hypertensive rats (SHR) supplemented with testosterone. This model displays all key features of BPH/LUTS i.e. prostate enlargement, increased sympathetic tone of bladder outlet mimicking the static and the dynamic components of voiding symptoms related to BPH and signs of detrusor overactivity. In addition, correlation between bladder wall or detrusor wall thickness and bladder outlet obstruction and symptom severity has been reported in BPH/LUTS patients [9, 10], thus histological assessment of detrusor wall area, sandwiched between the mucosa and adventitia, was performed in this study.

## Methods

Adult 8-week-old male Wistar Kyoto rats (WKY, *n* = 10) and SHR rats (SHR, *n* = 20) (Janvier labs, France) were used in this study. The number of animals used is the minimum that is consistent with scientific integrity and regulatory acceptability, consideration having been given to the welfare of individual animals in terms of the number and extent of procedures to be carried out on each animal. The animals were housed 1 week prior to the beginning of the experiments at the animal facility with free access to standard chow (Chow M20, 841,201, SDS, UK) and water and maintained on inversed 12 h dark/light cycle (light off 10:00/22:00). Beginning with the acclimatization period and during the whole protocol, the rats were housed by 2 in a ventilated cage (Allentown Inc., USA). The rat environment was enriched with kraft paper fibers for nesting, polycarbonate square tunnel and gnawing material (*SERLAB, France*). All procedures were approved by the local ethical committee (CEE47) and performed in accordance with the legislation on the use of laboratory animals (NIH publication N°85–23, revised 1996) and Animal Care Regulations in force in France as of 1988 (authorization from competent French Ministry of Agriculture - Agreement No. B78–423-1, July 2017). After the acclimation period, SHR rats were identified with a unique identification number; then using random number table, they were randomly allocated to 2 experimental groups receiving either vehicle, methylcellulose 5% (SHR-T, *n* = 10, mean weight prior to treatment 269.9 ± 4.1 g) or silodosin at 0.1 mg/kg/day (SHR-T + silodosin, n = 10, mean weight prior to treatment 267.8 ± 3.9 g) by oral gavage for 6 weeks. Concomitantly, SHR rats received daily subcutaneous testosterone supplementation at 3 mg/kg/day to induce prostate growth. WKY rats (WKY, *n* = 10, mean weight prior to treatment 250.2 ± 5.5 g) received daily subcutaneous sesame oil and vehicle by oral gavage.

At the end of the treatment period, cystometry experiments were performed. Finally, the rats were anesthetized with isoflurane (2.0–2.5%, Centravet, France) and then euthanized by intraperitoneal injection of pentobarbital at 0.1 ml/100 g of body weight (Centravet, France) in accordance with Animal Care Regulations in force in France. The bladder was then harvested, weighed and paraffin-embedded for histological examination and measure of the bladder wall thickness. The prostate was also harvested and weighed.

### Intravesical catheter implantation

As described previously [8], an intravesical catheter was implanted in the bladder dome of each rat 2 days before the cystometry experiments. Briefly, the rats were anesthetized with isoflurane (1.5–2.0%, Centravet, France) and their temperature maintained at 37 °C using a homeothermic blanket. Then a polyethylene catheter (PE-50: 0.965 mm OD) prefilled with saline solution was inserted through the apex bladder dome exposed via a midline abdominal incision. The catheter was fixed with a 4-zero cotton purse-string suture and its free end was tunneled subcutaneously and exteriorized at the back of the neck. Each rat was maintained individually in a cage with food and water ad libitum until cystometry experiment.

### Cystometry experiments

**Fig. 1:**
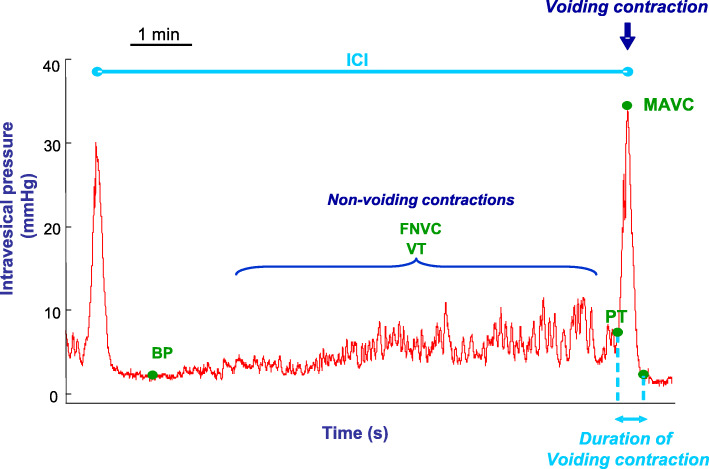
Illustration of the parameters computed for bladder voiding and non-voiding contractions during cystometry in a conscious of SHR-testosterone treated rat. MAVC: maximal amplitude of voiding contractions, BP: baseline intravesical pressure, PT: micturition pressure threshold, ICI: intercontraction interval. FNVC: frequency of non-voiding contractions, VT: volume threshold to elicit non-voiding contractions for each micturition cycle during the bladder filling phase

Cystometric investigations were performed in conscious rats. As previously described [8], the rats were placed in metabolic cages and the free tip of the bladder catheter was connected to a pressure transducer for bladder pressure monitoring and to a syringe pump for bladder perfusion. The rats were allowed an acclimation period then the bladder was continuously perfused with saline at 50 μl/min. Weighing devices placed underneath the metabolic cages were used to quantify the micturition volumes. Once the bladder continuous perfusion started, a 60-min stabilization period was considered to ensure reproducible micturition cycles i.e., the variations in micturition volumes and in intervals between micturition did not exceed ±10%, before starting a 60-min evaluation period. During this evaluation period, intravesical pressure was recorded and the following parameters related to voiding contractions were measured: maximal amplitude, baseline intravesical pressure, micturition pressure threshold and intercontraction interval for reflex-evoked bladder micturition. Also, frequency of non-voiding contractions and the volume threshold to elicit non-voiding contractions for each cycle during the filling phase were measured (Fig. 1). Furthermore, the infused volume between two voiding contractions, index of the bladder capacity, and the voided volume of each voiding contraction were analyzed, and the mean urine flow rate was assessed.

### Histological examination of bladder wall hypertrophy

The rats were euthanized and the bladder was harvested, paraffin embedded and 5-μm sections were performed using a microtome (RM 2245®, Leica Biosystems, France). The sections were stained with hematoxylin-eosin using an automated multistainer (ST5020® &CV5030 Coverslipper® Leica Biosystems, France) for evaluation of detrusor wall area, the detrusor being sandwiched between the mucosa and adventitia. The acquisition of slides images was then performed using Aperio AT2 - Scanner for On-Screen Diagnosis® (Leica Biosystems, France). Then images were analyzed with NIS Elements Software (NIS elements, Nikon, France) to compute the detrusor wall area from each section by a blinded experimenter. For each animal, 4 transverse sections were analyzed and a mean area of sections per animal was then determined.

### Statistical analysis

All results are presented as mean ± SEM. The number of animals included in the analysis were *n* = 10/10 for WKY and *n* = 7–8/10 for each SHR rats supplemented with testosterone group i.e., SHR-T and SHR-T + silodosin. The cystometry data of some rats were not interpretable due to either technical problem or urine emission by dribbling during cystometry experiments.

*P* values < 0.05 were considered significant. Statistical analyses were performed using Student’s t-test.

### Drugs and chemicals

Silodosin was dissolved in 0.5% methylcellulose. Silodosin was provided by Recordati (Italy). All other drugs and chemicals were purchased from Sigma-Aldrich (France).

## Results

There was no statistical difference on maximal amplitude of voiding contractions and baseline intravesical pressure between WKY and SHR-T. Conversely, all other parameters characterizing the voiding contractions were significantly decreased in SHR-T compared to WKY, respectively, 5.8 ± 0.5 mmHg versus 8.4 ± 0.8 mmHg for pressure threshold, *p* < 0.05 Student’s t-test; 615.4 ± 55.4 s versus 825.8 ± 67.7 s for intercontraction interval, *p* < 0.05 Student’s t-test; 512.8 ± 46.1 μL versus 688.2 ± 56.4 μL for infused volume as an index of bladder capacity, *p* < 0.05 Student’s t-test; and 19.5 ± 3.7 μL/s versus 35.8 ± 5.0 μL/s for mean urine flow rate, p < 0.05 Student’s t-test. Only the decrease in voided volume in SHR-T did not reach statistical significance compared to WKY (respectively, 523.4 ± 56.2 μL versus 699.0 ± 72.5 μL, *p* = 0.09 Student’s t-test). Furthermore, the frequency of non-voiding contractions was significantly increased and the volume threshold to elicit non-voiding contractions for each micturition cycle during the bladder filling phase was significantly decreased in SHR-T compared to WKY (respectively, 1.6 ± 0.1 number of NVC/min versus 1.2 ± 0.1 number of NVC/min for frequency, *p* < 0.05 Student’s t-test; and 33.1 ± 6.6% versus 61.8 ± 6.8%, *p* < 0.01 Student’s t-test).

**Fig. 2:**
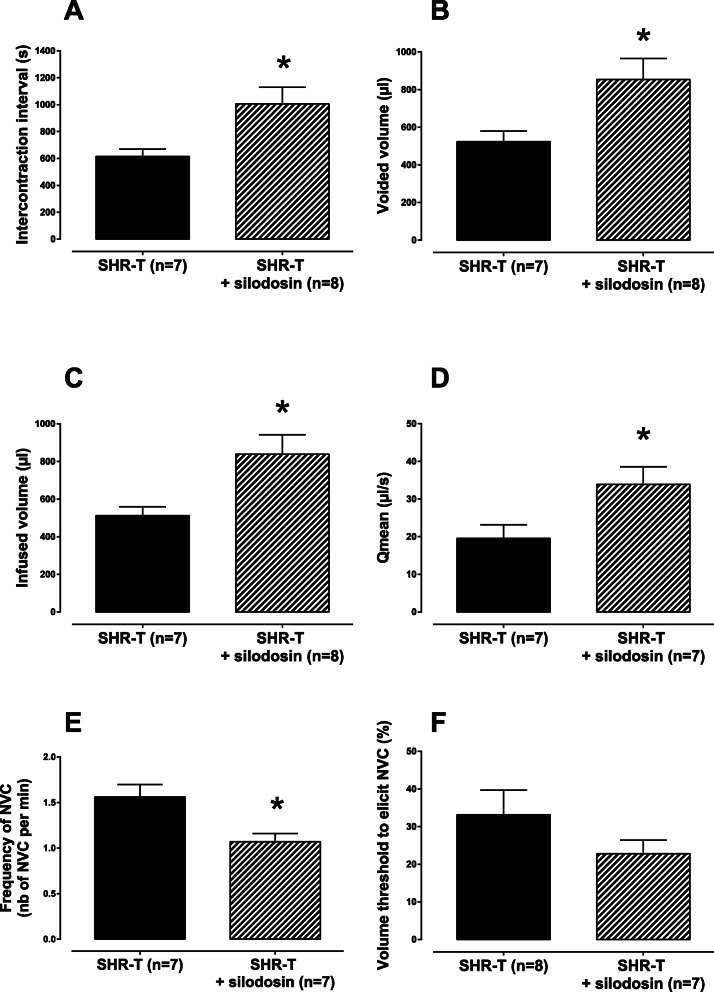
Cytometry parameters measured in conscious SHR supplemented by subcutaneous injections of testosterone that received by oral gavage once daily either vehicle (SHR-T) or silodosin at 0.1 mg/kg (SHR-T + silodosin) for 6 weeks. Data are expressed as mean ± SEM. *p < 0.05, Student’s t-test

Chronic administration of silodosin at 0.1 mg/kg/day for 6 weeks improved the voiding behavior of SHR-T by significantly increasing the intercontraction interval (*p* < 0.05 Student’s t-test, Fig. 2a), the voided volume (*p* < 0.05 Student’s t-test, Fig. 2b), the infused volume as an index of the bladder capacity (*p* < 0.05 Student’s t-test, Fig. 2c) and the mean flow rate (*p* < 0.05 Student’s t-test, Fig. 2d). Silodosin did not alter however the micturition pressure threshold. Compared to the vehicle-treated SHR-T, the silodosin-treated SHR-T showed a significantly lower number of non-voiding contractions (Fig. 2e) whereas the decrease in the volume threshold to elicit these non-voiding contractions did not reach statistical significance (Fig. 2f).

At a microscopic level, distinct layers were observed in bladder cross sections. The bladder lumen was lined by the urothelium that interdigitated with the lamina propria. The detrusor muscular area lied beneath the lamina propria and was surrounded by the most outer adventitia layer. Morphometric analysis showed a hypertrophy of the detrusor wall reflected by a 15% increase in the detrusor wall area of SHR-T compared to WKY (*p* < 0.05, Student’s t-test). Daily oral gavage of silodosin at 0.1 mg/kg for 6 weeks did not alter the increased bladder detrusor area of SHR-T.

SHR-T displayed a significant increase in prostate and bladder weights compared to WKY, respectively, 310.9 ± 19.8 mg/100 g body weight versus 162.5 ± 10.2 mg/100 g body weight for prostate, *p* < 0.001 Student’s t-test and 60.1 ± 2.9 mg/100 g body weight versus 48.4 ± 2.6 mg/100 g body weight for bladder *p* < 0.01 Student’s t-test. Silodosin administered at 0.1 mg/kg/d for 6 weeks did not modify either organ weights.

## Discussion

The effect of chronic treatment with silodosin on the urodynamic consequences of testosterone supplementation in the SHR, a model of BPH has been investigated. Whether the beneficial effect of silodosin on urodynamic parameters could be related to a drug effect on detrusor histology has also been assessed.

The relevance of the SHR supplemented with testosterone as a model for lower urinary tract symptoms resulting from BPH [8] is confirmed by the present study. Indeed, the SHR supplemented with testosterone i) showed prostate enlargement, a diagnostic criterion for human BPH and ii) displayed bladder hyperactivity characterized by a decrease in intercontraction interval, bladder capacity, voided volume and average voiding flow rate along with an increase in the frequency of non-voiding detrusor contractions during the filling phase. These urodynamic changes, along with the hypertrophy of the detrusor wall associated with mechanical obstruction caused by prostate enlargement and a previously described increase in urethral and prostate smooth muscle tone in SHR [11] mimics bladder dysfunction in symptomatic BPH/LUTS patients.

Silodosin, a highly selective α1A receptor antagonist, first-line treatment for BPH/LUTS, has been reported to improve storage and voiding symptoms in clinical trials [5, 6, 12, 13]. In different rat models with abnormal bladder function unrelated to prostate enlargement i.e., the SHR [14–16] and the bladder outlet obstruction models [17–19], silodosin was found to exert a beneficial effect on bladder function. Although the size of the prostate is not a predictive factor for the severity of LUTS, enlarged prostate is an important feature for a model of BPH/LUTS. Hence, it was important to investigate whether silodosin could improve bladder function in a model that displays key features of BPH i.e. prostate enlargement, increased sympathetic tone of bladder outlet mimicking the static and the dynamic components of BPH related voiding symptoms and detrusor overactivity.

Chronic silodosin treatment relieved storage symptoms in SHR-T as shown by the increased intercontraction interval and improved bladder capacity in silodosin-treated compared to vehicle-treated SHR-T. An increase in the voided volume and average voiding flow rate were also found, both parameters being related to the voiding phase of the micturition cycle.

Bladder hyperactivity is also characterized by non-voiding contractions, corresponding to non-voiding detrusor contractions during the filling phase [8, 14, 19]. Interestingly, silodosin decreased the frequency of non-voiding contractions in SHR-T. Such an effect of silodosin could neither be correlated to significant effects of the drug on prostate and/or bladder weight nor on any histological changes in the bladder.

We postulate that the beneficial effects of silodosin on storage symptoms could be due to an inhibitory effect by silodosin on bladder afferents activity triggering the micturition reflex. Such a hypothesis is supported by recent findings by Aizawa et al. [17], describing an inhibitory effect elicited by a 6-day constant infusion of silodosin on single–unit activities of mechanosensitive afferent Aδ- and C-fibers during the filling-phase in a bladder outlet obstruction rat model. Moreover, Yazaki et al., [20] evidenced that a constant infusion of silodosin for 2 weeks significantly reduced c-Fos expression in the spinal cord from rats with bladder outlet obstruction obtained by partial ligation of the urethra, c-Fos increased expression being an indicator of increased afferent input from mechanoreceptors in the lower urinary tract [21]. These authors concluded that the potential inhibitory effect of silodosin on bladder afferents was independent of a decrease in urethral resistance since silodosin did not decrease the bladder contraction pressure using the same model.

To date, α1A receptor antagonists are thought to relieve LUTS by reducing prostatic tone and urethral resistance via the inhibition of α1 receptor-mediated smooth muscle contractions. The molecular mechanisms of disease progression and bladder remodeling in humans have been recently reviewed [22] and several new mechanisms of action of α1 receptor antagonists, and more particularly of silodosin, an α1A receptor antagonist considered to have a higher uroselectivity than other α1A receptor antagonists, have been suggested. The impairment in bladder blood flow, subsequent to the bladder wall hypertrophy caused by bladder outlet obstruction, seems to play a key role in the natural history of BPH. The decrease in bladder blood flow due to ischemia coupled with an increased expression of mRNA of α1A receptors in bladder microvessels in a bladder outlet obstruction rat model [18] and SHR [16, 23] have been reported. Thus, silodosin could improve bladder blood flow in addition to its inhibitory effect on bladder afferents. Indeed, constant infusion of silodosin for 2 weeks improved bladder blood flow in a bladder outlet obstruction rat model while preventing an increase in voiding frequency [18]. Likewise, 6 weeks of silodosin oral delivered improved bladder blood flow while decreasing micturition frequency and voided volume in SHR [16, 23]. Masuda et al., [24] even suggested that the bladder ischemia induced by bladder outlet obstruction increases the generation of reactive oxygen species activating a signalling cascade leading to the stimulation of bladder afferent fibers. Finally, Goi et al., [25] suggested that silodosin may improve bladder blood flow and bladder contraction by preventing fibrosis of bladder smooth muscle in a model of atherosclerosis-induced chronic bladder ischemia. Thus, it would be interesting to investigate in future studies whether morphological remodeling by decreasing fibrosis of bladder smooth muscle in this model may account for the observed effect of silodosin on urodynamic parameters.

## Conclusions

This is a proof-of concept experimental study that described the beneficial effect of silodosin treatment on relieving the urodynamic consequences in an animal model, the testosterone supplemented SHR, a model of LUTS related BPH, suggesting an inhibitory effect of silodosin on the bladder afferent pathway. This effect could not be related to significant effects of the drug on prostate and/or bladder weight nor on any histological changes in the bladder. It should be noted, the cystometry technique in conscious rats used in this study, even though broadly used in experimental animal studies [14, 15, 17, 26, 27] to evaluate urodynamic parameters in conscious animals, is an invasive technique and not complemented with bladder electromyography test, two contrasting features compared to clinical setting. Future studies are needed to fulfill the limitations of this proof of concept study mainly (i) determining sample size using statistical method based on the current results and the expected loss of study individuals in different experimental endpoints and (ii) evaluating fibrosis by histological quantification and consequently futures studies would allow to investigate the effect of silodosin on morphological remodeling and fibrosis of bladder smooth muscle in this model of SHR supplemented with testosterone.

## Data Availability

Not applicable.

## Abbreviations

SHR: Spontaneously hypertensive rat
WKY: Wistar Kyoto rat
SHR-T: Spontaneously hypertensive rat supplemented with testosterone
SHR-T + silodosin: Spontaneously hypertensive rat supplemented with testosterone that received chronic silodosin treatment
LUTS: Lower urinary tract symptoms
BPH: Benign prostate hyperplasia
MAVC: Maximal amplitude of voiding contractions
BP: Baseline intravesical pressure
PT: Micturition pressure threshold
ICI: Intercontraction interval
NVC: Non-voiding contractions
FNVC: Frequency of non-voiding contractions
VT: Volume threshold to elicit non-voiding contractions for each micturition cycle during the bladder filling phase
